# 4,6,7,9,10,12,13,15-Octa­hydro-2*H*-1,3-dithiolo[4,5-*i*][1,4,7,12]dioxadithia­cyclo­tetra­decine-2-thione

**DOI:** 10.1107/S1600536809029468

**Published:** 2009-07-31

**Authors:** Rui-Bin Hou, Bao Li, Tie Chen, Bing-Zhu Yin, Li-Xin Wu

**Affiliations:** a Key Laboratory of Organism Functional Factors of Changbai Mountain, Yanbian University, Ministry of Education, Yanji 133002, People’s Republic of China; b State Key Laboratory of Supramolecular Structure and Materials, College of Chemistry, Jilin University, Changchun 130012, People’s Republic of China

## Abstract

In the title mol­ecule, C_11_H_16_O_2_S_5_, the two S atoms from the macrocycle are situated on opposite sides of the mean plane of the five-membered ring, deviating from it by 1.288 (3) and 1.728 (3) Å. In the crystal, weak inter­molecular C—H⋯S and C—H⋯O hydrogen bonds link the mol­ecules into layers parallel to (100). The crystal studied was a racemic twin.

## Related literature

For crown ether annulated 1,3-dithiol-2-thio­nes, see: Hansen *et al.* (1992 ▶); Trippé *et al.* (2002 ▶). For details of the synthesis, see: Chen *et al.* (2005 ▶). For a related structure, see: Hou *et al.* (2009 ▶)
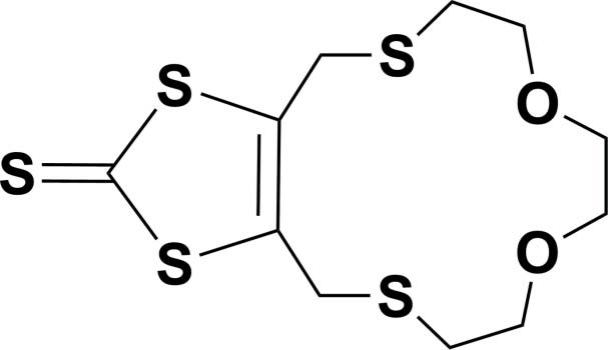


## Experimental

### 

#### Crystal data

C_11_H_16_O_2_S_5_
*M* *_r_* = 340.54
Monoclinic, 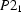

*a* = 8.9201 (18) Å
*b* = 8.5317 (17) Å
*c* = 10.128 (2) Å
β = 97.00 (3)°
*V* = 765.0 (3) Å^3^
*Z* = 2
Mo *K*α radiation
μ = 0.75 mm^−1^
*T* = 291 K
0.13 × 0.12 × 0.11 mm

#### Data collection

Rigaku R-AXIS RAPID diffractometer
Absorption correction: multi-scan (*ABSCOR*; Higashi, 1995 ▶) *T*_min_ = 0.909, *T*_max_ = 0.922
7527 measured reflections
3221 independent reflections
3100 reflections with *I* > 2σ(*I*)
*R*_int_ = 0.028

#### Refinement

*R*[*F* ^2^ > 2σ(*F* ^2^)] = 0.034
*wR*(*F* ^2^) = 0.088
*S* = 1.06
3221 reflections
164 parameters
1 restraint
H-atom parameters constrained
Δρ_max_ = 0.58 e Å^−3^
Δρ_min_ = −0.22 e Å^−3^
Absolute structure: Flack (1983 ▶); 1359 Friedel pairs
Flack parameter: 0.42 (9)

### 

Data collection: *RAPID-AUTO* (Rigaku, 1998 ▶); cell refinement: *RAPID-AUTO*; data reduction: *CrystalStructure* (Rigaku/MSC, 2002 ▶); program(s) used to solve structure: *SHELXS97* (Sheldrick, 2008 ▶); program(s) used to refine structure: *SHELXL97* (Sheldrick, 2008 ▶); molecular graphics: *PLATON* (Spek, 2009 ▶); software used to prepare material for publication: *SHELXL97*.

## Supplementary Material

Crystal structure: contains datablocks global, I. DOI: 10.1107/S1600536809029468/cv2592sup1.cif

Structure factors: contains datablocks I. DOI: 10.1107/S1600536809029468/cv2592Isup2.hkl

Additional supplementary materials:  crystallographic information; 3D view; checkCIF report

## Figures and Tables

**Table 1:** Hydrogen-bond geometry (Å, °)

*D*—H⋯*A*	*D*—H	H⋯*A*	*D*⋯*A*	*D*—H⋯*A*
C7—H7*A*⋯S1^i^	0.97	2.86	3.695 (3)	145
C10—H10*A*⋯O2^ii^	0.97	2.51	3.317 (3)	140

Symmetry codes: (i) 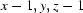; (ii) 
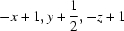
.

**Fig. 1.**
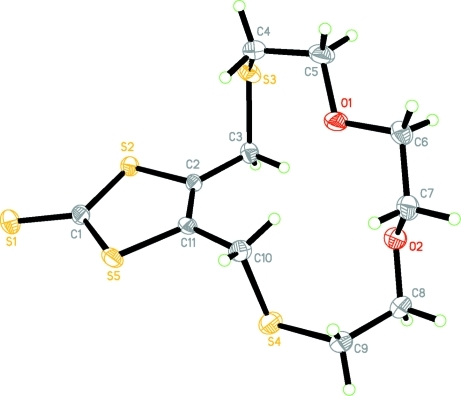
The molecular structure of the title compound showing the atomic numbering. Displacement ellipsoids of non-H atoms are drawn at the 30% probalility level.

## supplementary crystallographic information

### Comment

In the context of redox-responsive ligands, TTF is an ideal redox-active unit in view of its unique π-electron donating properties. Attachment of ligands such as crown ethers to TTF in many cases results in the electrochemical tunable ligands (Trippé *et al.*, 2002). Crowned 1,3-dithiole-2-thiones, important precursors to TTF derivatives, have also attracted attention (Hansen *et al.*, 1992). In this paper, we report the crystal structure of the title compound.

In the title compound (Fig. 1), all bond lengths and angles are normal and comparable with those reported for the related structure (Hou *et al.*, 2009). In the crystal, weak intermolecular C—H···S and C—H···O hydrogen bonds (Table 1) link the molecules into layers parallel to (*a+c*)*b* plane.

### Experimental

The title compound was prepared according to the literature (Chen *et al.*, 2005) and single crystals suitable for X-ray diffraction were prepared by slow evaporation a mixture of dichloromthane and petroleum (60–90 °C) at room temperatue.

### Refinement

Carbon-bound H-atoms were placed in calculated positions with C—H 0.97 Å and were included in the refinement in the riding model with *U*_iso_(H) = 1.2 *U*_eq_(C). The refined value of Flack parameter of 0.42 (9) suggests that the crystal studied was a racemic twin.

### Crystal data

**Table:**

C_11_H_16_O_2_S_5_	*F*(000) = 356
*M**_r_* = 340.54	*D*_x_ = 1.478 Mg m^−^^3^
Monoclinic, *P*2_1_	Mo *K*α radiation, λ = 0.71073 Å
Hall symbol: P 2yb	Cell parameters from 7211 reflections
*a* = 8.9201 (18) Å	θ = 3.1–27.5°
*b* = 8.5317 (17) Å	µ = 0.75 mm^−^^1^
*c* = 10.128 (2) Å	*T* = 291 K
β = 97.00 (3)°	Block, yellow
*V* = 765.0 (3) Å^3^	0.13 × 0.12 × 0.11 mm
*Z* = 2	

### Data collection

**Table:**

Rigaku R-AXIS RAPID diffractometer	3221 independent reflections
Radiation source: fine-focus sealed tube	3100 reflections with *I* > 2σ(*I*)
graphite	*R*_int_ = 0.028
ω scans	θ_max_ = 27.5°, θ_min_ = 3.1°
Absorption correction: multi-scan (*ABSCOR*; Higashi, 1995)	*h* = −11→11
*T*_min_ = 0.909, *T*_max_ = 0.922	*k* = −11→10
7527 measured reflections	*l* = −13→11

### Refinement

**Table:**

Refinement on *F*^2^	Secondary atom site location: difference Fourier map
Least-squares matrix: full	Hydrogen site location: inferred from neighbouring sites
*R*[*F*^2^ > 2σ(*F*^2^)] = 0.034	H-atom parameters constrained
*wR*(*F*^2^) = 0.088	*w* = 1/[σ^2^(*F*_o_^2^) + (0.0508*P*)^2^ + 0.1939*P*] where *P* = (*F*_o_^2^ + 2*F*_c_^2^)/3
*S* = 1.06	(Δ/σ)_max_ = 0.001
3221 reflections	Δρ_max_ = 0.58 e Å^−^^3^
164 parameters	Δρ_min_ = −0.22 e Å^−^^3^
1 restraint	Absolute structure: Flack (1983); 1359 Friedel pairs
Primary atom site location: structure-invariant direct methods	Flack parameter: 0.42 (9)

### Special details

**Table:**

Geometry. All e.s.d.'s (except the e.s.d. in the dihedral angle between two l.s. planes) are estimated using the full covariance matrix. The cell e.s.d.'s are taken into account individually in the estimation of e.s.d.'s in distances, angles and torsion angles; correlations between e.s.d.'s in cell parameters are only used when they are defined by crystal symmetry. An approximate (isotropic) treatment of cell e.s.d.'s is used for estimating e.s.d.'s involving l.s. planes.
Refinement. Refinement of *F*^2^ against ALL reflections. The weighted *R*-factor *wR* and goodness of fit *S* are based on *F*^2^, conventional *R*-factors *R* are based on *F*, with *F* set to zero for negative *F*^2^. The threshold expression of *F*^2^ > σ(*F*^2^) is used only for calculating *R*-factors(gt) *etc*. and is not relevant to the choice of reflections for refinement. *R*-factors based on *F*^2^ are statistically about twice as large as those based on *F*, and *R*- factors based on ALL data will be even larger.

### Fractional atomic coordinates and isotropic or equivalent isotropic displacement parameters (Å^2^)

**Table:**

	*x*	*y*	*z*	*U*_iso_*/*U*_eq_	
C1	0.9104 (3)	0.5403 (4)	1.0514 (2)	0.0344 (5)	
C2	0.6930 (2)	0.4126 (3)	0.8914 (2)	0.0264 (5)	
C3	0.5748 (3)	0.2952 (3)	0.8447 (2)	0.0342 (5)	
H3A	0.6206	0.1920	0.8492	0.041*	
H3B	0.5391	0.3164	0.7521	0.041*	
C4	0.3178 (3)	0.4733 (4)	0.8901 (3)	0.0406 (6)	
H4A	0.2524	0.4990	0.9568	0.049*	
H4B	0.3929	0.5556	0.8915	0.049*	
C5	0.2242 (3)	0.4741 (4)	0.7555 (3)	0.0445 (7)	
H5A	0.1638	0.3795	0.7439	0.053*	
H5B	0.1566	0.5637	0.7482	0.053*	
C6	0.2480 (3)	0.4848 (4)	0.5262 (3)	0.0426 (6)	
H6A	0.1704	0.5650	0.5179	0.051*	
H6B	0.2005	0.3842	0.5047	0.051*	
C7	0.3637 (3)	0.5190 (4)	0.4329 (3)	0.0418 (6)	
H7A	0.3150	0.5253	0.3421	0.050*	
H7B	0.4128	0.6185	0.4558	0.050*	
C8	0.5855 (3)	0.4162 (4)	0.3590 (3)	0.0427 (7)	
H8A	0.5382	0.4495	0.2722	0.051*	
H8B	0.6331	0.3156	0.3480	0.051*	
C9	0.7060 (3)	0.5337 (4)	0.4090 (3)	0.0470 (7)	
H9A	0.6589	0.6351	0.4175	0.056*	
H9B	0.7765	0.5438	0.3438	0.056*	
C10	0.6889 (3)	0.5696 (4)	0.6801 (2)	0.0381 (6)	
H10A	0.6885	0.6826	0.6690	0.046*	
H10B	0.5861	0.5322	0.6586	0.046*	
C11	0.7448 (2)	0.5293 (3)	0.8207 (2)	0.0290 (5)	
O1	0.3233 (2)	0.4825 (3)	0.65686 (18)	0.0432 (5)	
O2	0.4715 (2)	0.3969 (2)	0.44464 (17)	0.0385 (4)	
S1	1.03598 (9)	0.58767 (12)	1.17933 (8)	0.0558 (2)	
S2	0.78149 (7)	0.39272 (8)	1.05411 (6)	0.03282 (15)	
S3	0.41352 (8)	0.29178 (9)	0.93755 (8)	0.04456 (18)	
S4	0.80970 (8)	0.48036 (12)	0.56811 (7)	0.0539 (2)	
S5	0.89230 (7)	0.64009 (8)	0.90156 (7)	0.03733 (16)	

### Atomic displacement parameters (Å^2^)

**Table:**

	*U*^11^	*U*^22^	*U*^33^	*U*^12^	*U*^13^	*U*^23^
C1	0.0240 (11)	0.0456 (15)	0.0325 (11)	0.0034 (10)	−0.0003 (9)	−0.0064 (11)
C2	0.0195 (10)	0.0336 (13)	0.0252 (10)	0.0033 (8)	−0.0005 (8)	−0.0022 (9)
C3	0.0310 (12)	0.0346 (13)	0.0356 (12)	0.0012 (10)	−0.0020 (9)	−0.0022 (11)
C4	0.0330 (13)	0.0503 (17)	0.0400 (13)	0.0006 (12)	0.0105 (10)	−0.0063 (12)
C5	0.0259 (12)	0.0616 (19)	0.0473 (15)	0.0072 (12)	0.0094 (11)	0.0041 (14)
C6	0.0275 (13)	0.0597 (19)	0.0388 (13)	0.0049 (12)	−0.0030 (10)	0.0054 (13)
C7	0.0369 (14)	0.0520 (17)	0.0346 (12)	0.0011 (12)	−0.0028 (11)	0.0071 (12)
C8	0.0417 (15)	0.0582 (19)	0.0283 (11)	−0.0032 (13)	0.0048 (10)	0.0000 (12)
C9	0.0413 (15)	0.069 (2)	0.0313 (12)	−0.0104 (14)	0.0065 (11)	0.0044 (13)
C10	0.0318 (13)	0.0507 (16)	0.0315 (12)	0.0060 (11)	0.0028 (10)	0.0054 (11)
C11	0.0184 (10)	0.0390 (13)	0.0292 (11)	0.0012 (9)	0.0020 (8)	−0.0002 (10)
O1	0.0233 (8)	0.0716 (14)	0.0349 (9)	0.0071 (9)	0.0045 (7)	0.0047 (9)
O2	0.0346 (10)	0.0433 (11)	0.0377 (9)	−0.0016 (8)	0.0049 (7)	0.0018 (9)
S1	0.0336 (4)	0.0887 (7)	0.0416 (4)	−0.0110 (4)	−0.0092 (3)	−0.0101 (4)
S2	0.0298 (3)	0.0407 (3)	0.0267 (3)	−0.0002 (2)	−0.0015 (2)	0.0031 (2)
S3	0.0319 (3)	0.0508 (4)	0.0507 (4)	−0.0110 (3)	0.0041 (3)	0.0134 (3)
S4	0.0314 (3)	0.0975 (7)	0.0325 (3)	0.0086 (4)	0.0027 (3)	−0.0032 (4)
S5	0.0254 (3)	0.0466 (4)	0.0399 (3)	−0.0089 (3)	0.0031 (2)	0.0025 (3)

### Geometric parameters (Å, °)

**Table:**

C1—S1	1.656 (3)	C6—H6A	0.9700
C1—S2	1.708 (3)	C6—H6B	0.9700
C1—S5	1.730 (3)	C7—O2	1.413 (4)
C2—C11	1.341 (4)	C7—H7A	0.9700
C2—C3	1.489 (3)	C7—H7B	0.9700
C2—S2	1.747 (2)	C8—O2	1.424 (3)
C3—S3	1.812 (3)	C8—C9	1.511 (4)
C3—H3A	0.9700	C8—H8A	0.9700
C3—H3B	0.9700	C8—H8B	0.9700
C4—C5	1.510 (4)	C9—S4	1.815 (3)
C4—S3	1.805 (3)	C9—H9A	0.9700
C4—H4A	0.9700	C9—H9B	0.9700
C4—H4B	0.9700	C10—C11	1.490 (3)
C5—O1	1.414 (3)	C10—S4	1.824 (3)
C5—H5A	0.9700	C10—H10A	0.9700
C5—H5B	0.9700	C10—H10B	0.9700
C6—O1	1.409 (3)	C11—S5	1.741 (2)
C6—C7	1.510 (4)		
			
S1—C1—S2	124.03 (17)	C6—C7—H7A	110.0
S1—C1—S5	123.24 (18)	O2—C7—H7B	110.0
S2—C1—S5	112.70 (14)	C6—C7—H7B	110.0
C11—C2—C3	127.4 (2)	H7A—C7—H7B	108.4
C11—C2—S2	115.48 (18)	O2—C8—C9	113.8 (2)
C3—C2—S2	117.08 (18)	O2—C8—H8A	108.8
C2—C3—S3	114.98 (18)	C9—C8—H8A	108.8
C2—C3—H3A	108.5	O2—C8—H8B	108.8
S3—C3—H3A	108.5	C9—C8—H8B	108.8
C2—C3—H3B	108.5	H8A—C8—H8B	107.7
S3—C3—H3B	108.5	C8—C9—S4	113.3 (2)
H3A—C3—H3B	107.5	C8—C9—H9A	108.9
C5—C4—S3	115.9 (2)	S4—C9—H9A	108.9
C5—C4—H4A	108.3	C8—C9—H9B	108.9
S3—C4—H4A	108.3	S4—C9—H9B	108.9
C5—C4—H4B	108.3	H9A—C9—H9B	107.7
S3—C4—H4B	108.3	C11—C10—S4	110.08 (18)
H4A—C4—H4B	107.4	C11—C10—H10A	109.6
O1—C5—C4	108.3 (2)	S4—C10—H10A	109.6
O1—C5—H5A	110.0	C11—C10—H10B	109.6
C4—C5—H5A	110.0	S4—C10—H10B	109.6
O1—C5—H5B	110.0	H10A—C10—H10B	108.2
C4—C5—H5B	110.0	C2—C11—C10	125.7 (2)
H5A—C5—H5B	108.4	C2—C11—S5	116.22 (18)
O1—C6—C7	107.8 (2)	C10—C11—S5	118.0 (2)
O1—C6—H6A	110.1	C6—O1—C5	113.3 (2)
C7—C6—H6A	110.1	C7—O2—C8	113.1 (2)
O1—C6—H6B	110.1	C1—S2—C2	98.09 (12)
C7—C6—H6B	110.1	C4—S3—C3	103.16 (13)
H6A—C6—H6B	108.5	C9—S4—C10	99.88 (14)
O2—C7—C6	108.3 (2)	C1—S5—C11	97.47 (13)
O2—C7—H7A	110.0		

### Hydrogen-bond geometry (Å, °)

**Table:**

*D*—H···*A*	*D*—H	H···*A*	*D*···*A*	*D*—H···*A*
C7—H7A···S1^i^	0.97	2.86	3.695 (3)	145
C10—H10A···O2^ii^	0.97	2.51	3.317 (3)	140

Symmetry codes: (i) *x*−1, *y*, *z*−1; (ii) −*x*+1, *y*+1/2, −*z*+1.
